# Endoscopic Retrieval Technique of Proximally Migrated Pancreatic Stents: A Retrospective Study in a Tertiary Centre

**DOI:** 10.1155/2015/485980

**Published:** 2015-03-31

**Authors:** Yi Lu, Zheng Jin, Jia-chuan Wu, Li-ke Bie, Biao Gong

**Affiliations:** Digestive Endoscopy Center, Department of Gastroenterology, Ruijin Hospital, Shanghai Jiaotong University School of Medicine, Shanghai 200025, China

## Abstract

*Background*. There were scarce trials concerning the treatments and outcomes of proximal pancreatic stent migration. Herein, we did a retrospective study to discuss this problem from an endoscopist's point of view. *Patients and Methods*. From January 2009 to June 2014, patients with proximally migrated pancreatic duct stents were identified. Their clinical information was viewed. Retrieval techniques, success rates, and adverse events were analyzed. *Results*. A total of 36 procedures were performed in 34 patients; the median age of the patients was 53 years, with 17 males and 17 females. Eight patients' pancreatic duct stents could still be seen in the major or minor papilla and were pulled out with a snare forceps or a grasping forceps; in the remaining 28 procedures, the management was somewhat thorny; the retrieval called for several devices. Final success was achieved in 31 patients. No adverse event was observed in the process of ERCP procedures, 5 patients developed post-ERCP pancreatitis (PEP), 1 patient got infection, and 1 patient had haemorrhage. *Conclusions*. Endoscopic retrieval of migrated pancreatic stent is safe and less invasive; nonetheless, attention should be paid so as to reduce the incidence and degree of related adverse events, especially PEP.

## 1. Introduction

Pancreatic duct stents are used in a wide range of pancreatic diseases, including pancreatic ductal obstruction resulting from benign strictures or malignancy, drainage of pancreatic pseudocysts, symptomatic pancreaticobiliary malformations, pancreatic ductal stones, and prophylactic use for post-ERCP pancreatitis (PEP) [1–8]. With the increasing use of pancreatic duct stent, we experienced its merits, and, at the same time, its complications have also been recognized. Infection, pancreatitis, perforation, and stent migration are the common complications which we may encounter. Stent migrations can be divided into distal migration and proximal migration, and the former one is less harmful despite losing the power of drainage, while the latter possesses a more serious condition, due to a possibility of pancreatic duct damage. An early study [9] showed that the incidence of proximal migration was about 5.2%; however, till recently, there are scarce trials concerning the treatments and outcomes of proximal migration. Herein, we did a retrospective study to discuss this problem from an endoscopist's point of view.

## 2. Patients and Methods

### 2.1. Patients

We retrospectively reviewed all identified cases having proximally migrated pancreatic duct stents. Inclusion criteria were as follows: (1) admittance was between January 2009 and June 2014, (2) proximally migrated pancreatic duct stent was found, and (3) medical data could be obtained. Exclusion criteria were (a) pregnant woman and (b) ERCP procedure that was not done in our centre. Patients' basic information, laboratory tests, imaging results, ERCP operative details, and status after ERCP were viewed. Proximally migrated pancreatic duct stent was defined as the stent flap of duodenal side migrated into the pancreatic duct. Data analysis included indications for stenting, size of stent, length of stent, number of stents, symptoms presented since last procedure, retrieval techniques, success rates, degree of retrieval difficulty, and adverse events. The study protocol was approved by the institutional review board.

### 2.2. Endoscopic Retrograde Cholangiopancreatography Procedures

**Figure 1:**
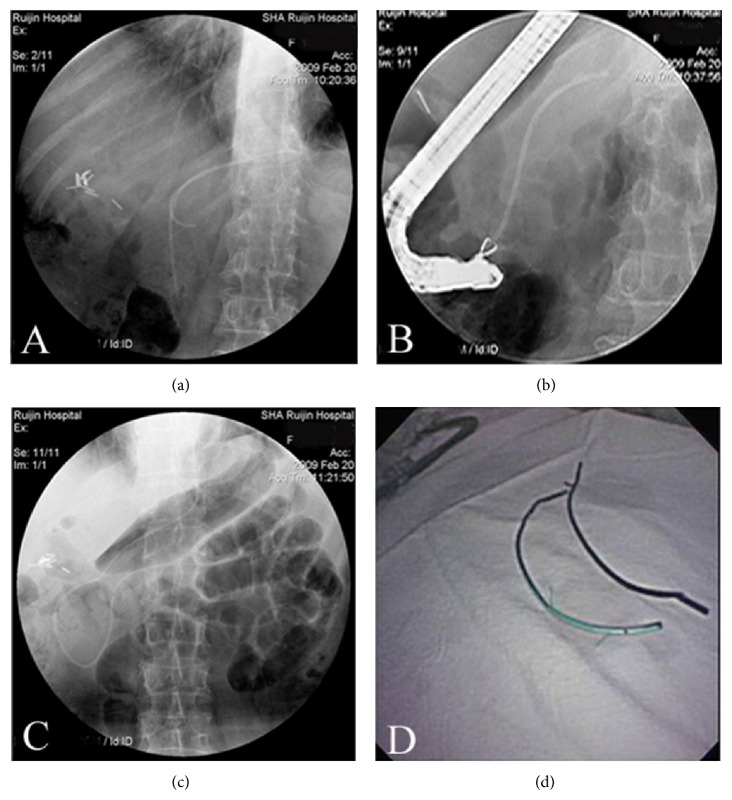
(a) Fluoroscopic view showing two pancreatic duct stents, with one migrated into the pancreatic duct. (b) Fluoroscopic view of stent extraction with grasping forceps after the tip of the stent has been pulled out of the papilla through balloon catheter. (c) Fluoroscopic view showing endoscopic nasopancreatic drainage has been performed. (d) Endoscopic demonstration of retrieved stent.

After the patient or the parent or guardian of each child had signed the informed written consent for ERCP procedure, patients were sedated with intravenous diazepam and/or pethidine or neither based on the anaesthetists' judgments. Before ERCP, a fluoroscopic view was obtained to confirm whether the pancreatic duct stent was still in. Then ERCP was performed using a side-viewing duodenoscope (TJF-260, JF-260, JF-240; Olympus Co., Ltd., Tokyo, Japan) by experienced endoscopists. If the tip of the stent could be seen in the papilla, a snare forceps (Alton Medical Instruments Co., Ltd., Shanghai, China) or a grasping forceps (Alton Medical Instruments Co., Ltd., Shanghai, China) was used to pull the stent out. In the situation where the stent was totally migrated into the pancreatic duct, a balloon catheter (Innovex Medical Co., Ltd., Shanghai, China) was used to pull the stent out of the papilla, and sometimes basket catheter (Extraction Basket; ENDO-FLEX Gmbh, Germany), forceps, or even metallic spiral stent retriever (Cook Medical) was needed (Figure 1 shows the process of migrated stent retrieval in a female patient). If direct traction or indirect traction had both failed, cannulating the stent before retrieval was required, and then a spiral retriever was used to fix the stent; with a pull of the retriever, the stent was also moved out. In case the working space was restricted by the strictures or sphincter, endoscopic sphincterotomy (EST) or endoscopic papillary balloon dilation (EPBD) was performed so as to make the retrieval possible. Adverse events of ERCP were assessed according to Cotton's Criteria [10].

### 2.3. Statistical Analysis

SAS version 8.2.0 (SAS Institute Inc., Cary, NC, USA) were used to perform the statistical analyses; continuous variables with normal distribution were presented with mean (range), and otherwise we used median (range). Categorical variables were presented with number (percentage).

## 3. Results

A total of 36 procedures were performed in 34 patients, 1 patient required 2 procedures, and 1 patient had experienced 2 times of migration requiring 2 procedures. The median age of the patients was 53 years (range 4–90 yrs old), with 17 males and 17 females. Indications for their previous endoscopic retrograde pancreatic drainage (ERPD) were pancreatic duct strictures or stones relating to chronic pancreatitis (29 patients, among whom 5 patients were also complicated with pancreas divisum, 2 patients with abnormal pancreatic ducts), pancreas divisum (1 patient without chronic pancreatitis), anomalous pancreaticobiliary ductal junction (2 patients), recurrent acute pancreatitis (3 patients), and pancreatic pseudocyst (1 patient). Sizes and lengths of their previous placed stents were presented in Table 1 (5 patients' information about their previous ERPD was not sufficient). Since the previous ERPD, 16 patients presented abdominal pain, 2 patients had an episode of acute pancreatitis, 3 patients had other symptoms (fever, abdominal discomfort), and 13 patients almost had no symptoms until the migration was detected in this process of ERCP. The median time for the development of symptoms since last ERPD was 6 months (range 1 month to 24 months), and the median time for the detection of stents migration since last ERPD was 6 months (range 1 month to 33 months). Fourteen patients had had endoscopic sphincterotomy (EST) before this procedure, 1 patient had had endoscopic papillary balloon dilation (EPBD), and 1 patient had had precut.

During the 36 procedures, 8 patients' pancreatic duct stents could still be seen in the major or minor papilla and were pulled out with a snare forceps or a grasping forceps without difficulty; in the remaining 28 procedures, the tip of the stents could not be seen, and the management was somewhat thorny. In one patient, we failed to cannulate the catheter into the pancreatic duct, and, in an eleven-year-old child, who refused to cooperate, the retrievals were prevented. For the rest, 5 patients needed EST or endoscopic papilla sphincterotomy (EPS) to enlarge the orifice so as to facilitate retrieval; 9 patients needed EPBD to enlarge the orifice or the cramped pancreatic duct. In 3 procedures, though various attempts had been tried, the stents still could not be moved out. Altogether in 31 procedures, the migrated stent was retrieved successfully. (Table 2 summarizes the success rates in each stent removal step.)

After retrieval of the migrated stents, 11 patients had one stent placed in their pancreatic duct again (among whom 4 patients were through minor papilla), 4 patients had two stents, and 17 patients received endoscopic nasopancreatic drainage (ENPD) to improve the pancreatic ductal drainage.

No adverse event was observed in the process of ERCP procedures. 5 patients developed PEP; Table 3 shows the characteristics of the patients who developed PEP. One patient got biliary infection and recovered with the administration of antibiotics. Another patient had hematemesis and bloody fluid in the ENPD duct; after hemostasis therapy and fluid infusion, the haemorrhage soon was stopped. All of the adverse events were in mild or moderate degree.

## 4. Discussion

With increased use of pancreatic duct stents, its adverse events have also become recognized. In 1992, Johanson et al. [9] reported that the incidence for distal and proximal pancreatic stent migration was 7.5% and 5.2%, respectively. And they further pointed out that malignant strictures, larger diameter stents, sphincter of Oddi dysfunction, and longer stents were associated with proximal pancreatic stent migration. Comparatively, distal migration is rarely harmful, as the stent is excreted from the intestinal tract [11], while proximal migration is risk factor for acute pancreatitis and pancreatic duct damage. Symptoms may force the patients to go to see the doctors and find the problem soon. However, sometimes it would be asymptomatic. In our study, more than one-third of the patients almost had no symptoms, and the migration was detected when they go to our hospital to have reexamination 6 months or so after the previous ERCP, as we usually ask the patients to have reexamination or stent exchange 3 to 6 months after the first ERCP, and the time was adjusted by the specific situations. And this asymptomatic state may last for a certain period of time. Lahoti et al. [12] followed up on 5 asymptomatic patients with migrated stents for 1 year to 5 years, and no complication was reported.

Proximally migrated stent can be retrieved through endoscopic or surgical method; generally speaking, endoscopic retrieval seems to be more favourable, since it is much less invasive when compared with surgery. We would take surgical means into consideration if endoscopic retrieval had failed after several attempts, or the patient was complicated with other complex conditions which also needed surgery. After a thorough search in PubMed, just several articles describing endoscopic retrieval of the migrated pancreatic stent were found. The technical success rates range from 77% to 100% [11–14], and our study is in consistency with the results. The procedure for endoscopic retrieval of migrated stents has been performed depending on the ingenuity of individual endoscopists [14]. The retrieval techniques can be classified mainly into 3 methods: (i) direct traction using various devices, (ii) indirect traction with a balloon catheter, and sometimes aided by direct devices, and (iii) cannulating the pancreatic stent lumen before retrieval [11, 12, 15]. From our experience, if the tip of the migrated stents could be seen in the major or minor papilla, we would choose direct traction; otherwise, a balloon catheter was first used to indirectly tract the stent out of the papilla and then a snare forceps, a grasping forceps, or a basket forceps was used to pull the stent directly. In 2 patients, both direct and indirect methods have failed; then we use the guide-wire and cannulated it into the pancreatic stent lumen; later a 7 Fr metallic spiral retriever was used to fix and pull the stent out eventually. Tarnasky et al. indicated that the difficulty of endoscopic retrieval had something to do with the migration upstream of a stenotic region in the biliary tract, and this suits the pancreatic duct well. On the imaging of pancreatogram, a stenosis could be observed easily, and EPBD or bouginage was performed to dilate the stenotic part, so as to provide enough space for retrieval.

But sometimes things just do not go as we expected; possibilities are that though various means had been tried, the stent still could not be retrieved endoscopically. According to the previous research [13], failure for stent removal was most associated with downstream stenosis (4 of 5 cases); other reasons were repeat ERCP, stent impaction, duct edema, or stent fragmentation. In our study, except for the above reasons, changed anatomical position due to pancreaticojejunostomy, derangement of the pancreatic head duct, a sharp angle of the pancreatic duct caused by the migrated stents, and patient's noncooperation were also responsible for the failure. Some experts [16] held that if endoscopic retrieval was in vain, then surgery is required, or a second stent should be placed. In our 5 failed cases, we performed ERPD in 4 patients (the uncooperative patient did not have stent placed, and one patient had 2 stents). In fact, careful follow-up for patients without symptoms may be an option, especially for those with high risk of surgery [13]. And a supplement of ERPD drainage while leaving the first alone may create condition for next successful removal. Take the case numbered 22 and 23 for example, who was actually the same patient. We failed at first attempt and left a stent to improve the drainage; eight months later, we had a second trial, and this time we succeeded.

Retrieving the migrated stents endoscopically, though much less invasive, can still bring about adverse events. Price et al. reported pancreatic duct disruption with subsequent leakage, stent fragmentation, and PEP, each having occurred in 1 of 23 patients [13]. In this study, the main adverse event was PEP, with 5 in 36 procedures, and secondly was infection and haemorrhage. Compared with the former two studies conducted by us (the first compared the safety and efficacy of EST and EPBD in common bile duct stones removal, and the second evaluated the use of ERCP in pancreas divisum), it was apparent that the incidence of PEP for migrated pancreatic duct stent retrieval was higher than those in the previous two studies, and the difference in the incidence of infection or haemorrhage was not that obvious. Theoretically, stent removal may do damage to the pancreatic duct, so as to trigger PEP, and placement of ERPD or ENPD would be ideal to prevent PEP [11, 13]. Price et al. performed ENPD in 15 of 22 patients (68%), and 2 cases had adverse events (9.1%) [13]; Matsumoto et al. performed ENPD in 4 of 5 patients (80%), and only one patient had PEP (20%) [11]. In our study, 15 patients (41.67%) received ERPD, and 17 patients (47.22%) had ENPD; the choice of ERPD or ENPD was mainly based on whether strictures or stones existed in the pancreatic duct, which needed prolonged stent drainage. On the other hand, the procedures should be performed by experienced endoscopists with caution so as to diminish pancreatic duct stimulations and injuries.

Though the incidence of pancreatic duct stent proximal migration is not high, the consequence is unpredictable; it can be asymptomatic or cause ductitis, stent occlusion, pancreatitis, and so forth [13, 17, 18]. The specific probability for the above is not clear for now. In uncomplicated cases, endoscopic intervention is a good choice, while for the complicated or failed cases after endoscopic intervention, pros and cons should be balanced, so as to obtain the best outcome.

Our study was restricted by its retrospective and single-centred nature; prospective trials with great sample sizes and control group are needed. In conclusion, the symptoms of pancreatic duct stents migration may be various, reexamination is recommended, and most of the migration can be retrieved by endoscopic means, though various forceps and even retriever are needed. In general, endoscopic retrieval is safe and less invasive; nonetheless, attention should be paid so as to reduce the incidence and degree of related adverse events, especially PEP.

## Figures and Tables

**Table 1:** List of the patients undergoing ERCP for migrated pancreatic stents retrieval.

Number^∗^	Age	Sex	Indication	Size	Length	EST	Devices	Papilla	Success
1	83	M	CP	7 Fr	9 cm	—	Balloon, forceps	ma	Y
2	55	M	CP	?	?	EPBD	Balloon, basket	ma	N
3	25	M	CP, PD	7 Fr	9 cm	—	Forceps	min	Y
4	47	M	CP	7 Fr	9 cm	EST	Balloon, forceps	ma	Y
5	16	M	CP	7 Fr	7 cm	—	Balloon, forceps	ma	Y
6	52	M	CP	8.5, 7 Fr	7 cm, 10 cm	—	Balloon	ma	Y
7	27	F	APBDJ	5 Fr	7 cm	EST	Forceps	ma	Y
8	33	M	CP	8.5 Fr	10 cm	—	Balloon	ma	Y
9	31	F	CP, PD	7 Fr ∗ 2	7 cm (ma), 5 cm (min)	EST, EPBD	Balloon, forceps, and basket	min	Y
10	46	M	ARP	7 Fr	9 cm	—	Balloon, forceps	ma	Y
11	60	F	CP	7 Fr	9 cm	EPBD	Balloon, forceps, basket, and retriever	ma	Y
12	79	F	PD	5 Fr	9 cm	—	Balloon, forceps	ma	Y
13	60	M	CP	5 Fr	7 cm	—	Forceps, balloon, and basket	ma	Y
14	74	F	APBDJ	?	?	—	Balloon, forceps	ma	Y
15	23	F	CP, PD	?	?	EST	Basket	min	Y
16	54	M	CP	7 Fr	5 cm	—	Forceps	ma	Y
17	50	M	CP	7 Fr	7 cm	—	Forceps	ma	Y
18	50	F	CP	8.5 Fr, 7 Fr (ma) 6 Fr (min)	10 cm, 5 cm (ma) 6 cm (min)	—	Forceps	ma	Y
19	62	F	CP	7 Fr	9 cm	—	—	ma	N
20	38	M	ARP	?	?	—	Balloon, forceps, and basket	ma	Y
21	68	F	CP, APD	7 Fr	9 cm	EPBD	Balloon, forceps, basket, and retriever	ma	Y
22	13	F	CP, APD	5 Fr	7 cm	EST	Balloon, forceps, and basket	ma	N
23	14	F	CP	5 Fr	7 cm	—	Forceps	ma	Y
24	72	F	CP	?	?	—	Balloon, forceps	ma	Y
25	59	F	CP, PD	7 Fr	9 cm	EPBD	Basket, forceps	ma	Y
26	64	M	CP	5 Fr	7 cm	—	Forceps	ma	Y
27	31	F	CP	5 Fr	7 cm	EPBD	Balloon, forceps	ma	Y
28	33	F	CP	7 Fr	9 cm	EPBD	Balloon, forceps, and basket	ma	Y
29	12	M	CP	7 Fr	7 cm	—	Forceps	ma	Y
30	72	M	Pseudocyst	7 Fr	5 cm	—	Balloon, forceps	ma	Y
31	68	F	ARP	5 Fr	9 cm	—	Balloon, forceps	ma	Y
32	11	F	CP	5 Fr	7 cm	—	—	ma	N
33	90	M	CP	5 Fr	9 cm	—	Balloon, forceps	ma	Y
34	62	F	CP, PD	7 Fr	7 cm	—	Forceps	min	Y
35	60	F	CP	7 Fr ∗ 2	12 cm, 7 cm	EPBD	Balloon, forceps	ma	N
36	4	M	CP	7 Fr	5 cm	EPBD	Balloon, forceps	ma	Y

^∗^Numbers 22 and 23 were the same patient; numbers 27 and 28 were the same patient.

ERCP: endoscopic retrograde cholangiopancreatography; EST: endoscopic sphincterotomy; EPBD: endoscopic papillary balloon dilation; CP: chronic pancreatitis; PD: pancreas divisum; APBDJ: anomalous pancreaticobiliary ductal junction; ARP: acute recurrent pancreatitis; APD: abnormal pancreatic ducts; ma: major papilla; min: minor papilla; balloon: balloon catheter; forceps: snare forceps or grasping forceps; basket: basket catheter; retriever: metallic spiral stent retriever; Y: yes; N: no.

**Table 2:** Stent removal success rates in each step.

Step	Success rates
Direct retraction	87.50%
Indirect retraction	84.62%
Spiral retrieval	100%
Failed but with ERPD	80.00%

ERPD: endoscopic retrograde pancreatic drainage.

**Table 3:** Characteristics of the patients who developed PEP.

Number	Age	Sex	Indication	EST/EPBD	Devices	Success	ERPD/ENPD
2	55	M	CP	EPBD	Balloon, basket	N	ERPD
10	46	M	ARP	—	Balloon, forceps	Y	ENPD
15	23	F	CP, PD	EST	Basket	Y	ENPD
16	54	M	CP	—	Forceps	Y	ERPD
19	62	F	CP	—	—	N	ERPD

PEP: post-ERCP pancreatitis; EST: endoscopic sphincterotomy; EPBD: endoscopic papillary balloon dilation; CP: chronic pancreatitis; PD: pancreas divisum; ARP: acute recurrent pancreatitis; Y: yes; N: no.
